# Pathology, Practical Medicine, and Therapeutics

**Published:** 1838-07

**Authors:** 


### Selections from American and Colonial Journals.

### PATHOLOGY, PRACTICAL MEDICINE, AND THERAPEUTICS.

*Extraordinary Case of Electrical Excitement.* By Dr. Willard Hosford, of Orford, New Hampshire, United States.

A lady, of great respectability, during the evening of the 25th of January, 1837, the time when the aurora occurred,* became suddenly and unconsciously charged with electricity; and she gave the first exhibition of this power in passing her hand over the face of her brother, when, to the astonishment of both, vivid electrical sparks passed to it from the end of each finger. The fact was immediately mentioned; but the company were so sceptical that each, in succession, required, for conviction, both to see and feel the spark. On entering the room soon afterward, the combined testimony of the company was insufficient to convince me of the fact until a spark, three-fourths of an inch long, passed from the lady's knuckle to my nose, causing an involuntary recoil. This power continued with augmented force from the 25th of January to the last of February, when it began to decline, and became extinct by the middle of May. The quantity of electricity manifested during some days was much more than on others, and different hours were often marked by a light variableness; but it is believed that, under favorable circumstances, from the 25th of January to the 1st of the following April, there was no time when the lady was incapable of yielding electrical sparks.

* Speaking of it, Dr. Hosford adds, that "the heavens were lighted with a crimson aurora of such uncommon splendour as to excite no ordinary emotions in every observer."

The most prominent circumstances which appeared to add to her electrical power were, an atmosphere of about 80° Fahr., moderate exercise, tranquillity of mind, and social enjoyment: these, severally or combined, added to her productive power, while the reverse diminished it precisely in the same ratio. Of these a high temperature evidently had the greatest effect, while the excitement diminished as the mercury sunk, and disappeared before it reached zero. The lady thinks fear alone would produce the same effect, by its check on the vital action. We had no evidence that the barometrical condition of the atmosphere exerted any influence, and the result was precisely the same whether it were humid or arid. It is not strange that the lady suffered a severe mental perturbation from the visitation of a power so unexpected and undesired, in addition to the vexation arising from her involuntarily giving sparks to every conducting body that came within the sphere of her electrical influence; for, whatever of the iron stove or its appurtenances, or the metallic utensils of her work-box, such as needles, scissors, knife, pencil, &c. &c., she had occasion to lay her hands upon, first received a spark, producing a consequent twinge at the point of contact. The imperfection of her insulator is to be regretted, as it was only the common Turkey carpet of her parlour, and it could sustain an electrical intensity only equal to giving sparks one and a half inch long: these were, however, amply sufficient to satisfy the most sceptical observer of the existence, in or about her system, of an active power that furnished an uninterrupted flow of the electrical fluid; of the amount of which, perhaps, the reader may obtain a very definite idea by reflecting upon the following experiments:—When her finger was brought within one-sixteenth of an inch of a metallic body, a spark, that was heard, seen, and felt, passed every second. When she was seated with her feet on the stove-hearth (of iron), engaged with her books, with no motion but that of breathing and the turning of leaves, then three or more sparks per minute would pass to the stove, notwithstanding the insulation of her shoes and silk hosiery. Indeed, her easy chair was no protection from these inconveniences; for this subtle agent would often find its way through the stuffing and covering of its arms to its steel framework. In a few moments she could charge other persons insulated like herself; thus enabling the first individual to pass it on to a second, and the second to a third.

When most favorably circumstanced, four sparks per minute, of one inch and a half, would pass from the end of her finger to a brass ball on the stove: these were quite brilliant, distinctly seen and heard in any part of a large room, and sharply felt when they passed to another person. In order further to test the strength of this measure, it was passed to the balls by four persons forming a line: this, however, evidently diminished its intensity, yet the spark was bright. . . .

The lady had no internal evidence of this faculty,—a faculty *sui generis:* it was manifest to her only in the phenomena of its leaving her by sparks, and its dissipation was imperceptible, while walking her room or seated in a common chair, even after the intensity had previously arrived at the point of affording one and a half inch sparks. Neither the lady's hair or silk, so far as was noticed, was ever in a state of divergence; but, without doubt, this was owing to her dress being thick and heavy, and to her hair having been laid smooth at her toilet, and firmly fixed, before she appeared upon her insulator. . . .

This lady is the wife of a very respectable gentleman of this place: she is aged about thirty, of a delicate constitution, nervous temperament, sedentary habits, usually engaged with her books or needle-work, and generally enjoying a fine flow of spirits. She has, however, never been in sound health, but has seldom been confined to her bed by sickness even for a day. During the past two years she has suffered several attacks of acute rheumatism, of only a few days' continuance; but, during the autumn and the part of the winter preceding her electrical development, she suffered much from unseated neuralgia in the various parts of her system, and was particularly affected in the cutis vera, in isolated patches; the sensation produced being precisely like that caused by the application of water heated to the point a little short of producing vesication.

*Silliman's American Journal of Science. Jan.* 1838.

*On Purulent Discharges from the Bladder and Rectum in Hepatic Diseases in India.* By J. Mouat, m.d. Surgeon H. M. 13th Dragoons.

This is an important paper, containing a detail of thirteen cases of disease of the liver, chiefly hepatitis, in all of which there was discharge of pus from some of the natural passages, generally with relief, and in most without any direct communication existing between the original seat of the fluid and its vicarious outlet. The following table gives a general view of the results:

*Table of Purulent Discharges.*

| Cases. | Names. | Pus passed by | | | | Remarks. |
|---|---|---|---|---|---|---|
| | | Urine. | Stools. | Expectoration. | Vomiting. | |
| 1 | J. Ward ......... | 1 | 1 | 0 | 0 | Recovered. |
| 2 | T. Rippen ....... | 0 | 1 | 1 | 0 | Ditto, but died the 2d attack. |
| 3 | J. Gibson ....... | 1 | 1 | 1 | 0 | Died. |
| 4 | J. Young......... | 0 | 1 | 0 | 0 | Recovered. |
| 5 | R. Mallalew..... | 1 | 1 | 0 | 0 | Died. |
| 6 | G. Kennedy .... | 1 | 1 | 0 | 0 | Ditto. |
| 7 | G. Munnings ... | 1 | 0 | 0 | 0 | Recovered. |
| 8 | M. Reddy....... | 1 | 0 | 0 | 0 | Ditto. |
| 9 | M. Smith........ | 1 | 1 | 0 | 0 | Died. |
| 10 | J. Thomas ....... | 1 | 0 | 0 | 0 | Ditto. |
| 11 | H. Jackson ...... | 1 | 1 | 0 | 0 | Recovered. |
| 12 | M. Leamy ....... | 1 | 1 | 1 | 1 | Died. |
| 13 | C. Pearson ...... | 1 | 1 | 1 | 0 | Under treatment. |
| | Total......... | 11 | 10 | 4 | 1 | |

We extract two of the cases, one of recovery, the other fatal.

Case viii. Private Michael Reddy, ætat. forty. In India fifteen years. Admitted 12th July, 1834, with acute hepatitis, attended with nausea, vomiting, general weakness, loss of appetite, fever, &c. On the following day the side appeared enlarged and very tender on pressure. Like the other cases, was actively treated, yet the pain and swelling continued unabated till the 17th, when his mouth became affected from mercury, and at the same time a deposition of pus took place in the urine, with great relief to the pain, &c. and the swelling appeared somewhat reduced. From this period he continued to pass matter in his urine to the extent of two to three ounces in the twenty-four hours, till the beginning of September, when a gradual decrease of both pain and swelling, and an improvement in his health and strength took place. During September, pus was observed in less quantity, and about 20th November it ceased entirely; and on the 26th of that month he was discharged to his duty quite well, and is now enjoying good health.

Case ix. Private Mark Smith, ætat. twenty-three. In India one year. Admitted 18th June, 1834, with acute hepatitis, complicated with dysentery, slight fever, nausea, general debility, &c.; copiously bled, and actively treated, yet on the fifth day of admission his side became much swollen and very painful, and the following day pus was observed in his urine, with evident relief; the swelling was now considerably reduced, and there was slight ptyalism from the mercury he had taken. On the 24th the pain returned with great violence, (though he continued passing matter largely both by urine and stool,) followed by difficulty of breathing, weakness, copious cold perspiration, dyspnœa and lowness of spirits, &c., and he died on the 26th, or eight days after admission, in great agony.

*Dissection* disclosed several abscesses of various sizes in both lobes of the liver, some running into each other, and others quite distinct and filled with pus, but no communication could be traced either with the kidneys or intestines, though the urinary bladder appeared filled with a mixture of urine and purulent matter.

[We entirely agree with Dr. Mouat in his remarks on these cases. He says,] The deposits by stool, urine, and expectoration were examined in all the patients in the usual manner by the most approved tests, particularly the sulphuric acid, &c.; and though these be of a negative nature, still, when taken together with the appearance of the matter voided, left no doubt of its nature. Beside which, the previous symptoms in some, the swelling of the side in others, and the dissection in all the fatal terminations, are confirmative of the circumstance. One or two cases, selected to illustrate particular views, might create doubt, but there can be none where there were so many instances, many of them protracted, all closely watched by the other medical officers of the corps, and many of them viewed with great interest by professional friends; and where the dissections in the fatal cases were made by the assistants attached to the regiment, with the specific purpose of tracing, if possible, any direct channel of communication between those abscesses and the intestines, lungs, and kidneys.

*Calcutta Quarterly Medical Journal. July,* 1837.

---

*Case of Extra-Uterine Pregnancy.* By ALLAN WEBB, Esq.

WHEN Mr. Webb was summoned to see this patient, a Bengali woman, sixteen years of age and married, she had been ill three days, vomiting frequently, and was supposed to be dying of cholera. She was restless, tossing about in bed; no pulse, or an extremely feeble one. Respiration 60°: no pain in the abdomen upon pressure: no cholera expression of face. The abdomen was, however, enlarged, but soft, and yielding, no resistance being offered by the muscles to pressure. To enquiries if she were pregnant, she, and those around her, replied in the negative. I could not feel either uterus or bladder enlarged.

Venesection was performed. The blood, instead of being black, was scarcely deeper coloured than common serum; flowed slowly at first, more freely afterwards, and improved in colour. In raising herself to vomit she nearly fainted, and no more blood flowed from the arm. Gave a little ether, and ordered a warm bath to be prepared. She seemed somewhat easier after the ether: but in the act of raising her, to enter the bath, she breathed her last.

The body was examined three hours after death.

On puncturing the abdomen immediately below the sternum, blood gushed out. Before proceeding further, I suffered an immense quantity to escape, as much indeed as filled two wash-basins On fully laying open the cavity of the abdomen, all the organs appeared to be in health, but having the peculiar bleached appearance observed in slaughtered animals. The omentum which was adherent in the hypogastric regions being raised, and the bowels turned aside; the pelvis was seen to be filled up with a large and tolerably firm coagulum of black blood. Removing this, the bladder was observed contracted to the size of a small apple. The uterus of the natural size, but an enlargement was observed in the right fallopian tube, about the size of a large walnut, with which the large coagulum before spoken of was intimately connected. This enlargement was exceedingly thinned, anteriorly, with one or two black spots, ready to give way; whilst, superiorly, it had burst and given exit to a small fœtus, in which the rudiments of a spine, head and limbs, could be distinctly made out through the membranes. The placenta had not wholly escaped, part of it being still adherent to the internal surface of the fallopian tube. The free villous surface seeming to consist of the torn mouths of an immense congeries of small vessels, whence the hemorrhage had come. No larger vessel could be found to account for it, yet both fœtus, placenta, and membranes were not bigger than a good-sized walnut. This rent fallopian tube is intimately connected with the firmest portion of the coagulum. Right ovary, size of a fig, containing cysts: left ovary and fallopian tube healthy: uterus closed with tenacious secretion, lined with membrana decidua: vagina bleached and corrugated.

*Calcutta Quarterly Medical Journal. July,* 1837.

*On the Cure of Stammering by Moral Means.* By Edward Warren, m.d., of Boston (U. S.)

[We recommend the following very sensible observations to the attention of our readers. They are extracted from a well-written memoir on the general subject of stammering, which will well repay perusal. We regret that our limits do not admit of further extracts.]

I may be asked, if I maintain the importance of an experienced teacher: can parents who have children thus afflicted, do nothing themselves? I answer that they may do almost everything. With children, almost everything can be done by moral treatment; and according to the moral management they meet with, will the disease be confirmed or eradicated. The subjects are generally children of extreme nervous susceptibility and of feeble constitutions. The ordinary means for producing vigour and robustness, and for strengthening the nervous system must be resorted to. The muscular system must be developed as far as possible. If the chest is narrow and contracted, every means must be employed of bringing into action the muscles of the arms and chest. For this purpose gymnastic exercises, the use of dumb-bells, and various sports may be recommended. In this way, a great deal may be done to produce fulness of the chest. The child may be encouraged in the practice of these exercises as a means of acquiring physical strength, without his attention being called to his defect of speech. As soon as hfi is capable of reasoning, let him be driven as much as possible into the society of other children. If his defect is laughed at, let him be habituated to bear ridicule without flinching; let him be taught that those whose feelings allow them to ridicule defects or deformities, are much more worthy of pity than the subjects of those deformities. Let him be carried to the abodes of the deaf and dumb; teach him how much happier he is than they, how great a blessing he enjoys in the use of speech, even if his speech is imperfect. Carry him also into the presence of the blind, the lame, and the deformed. Let him be familiar with the sight of those who are greater sufferers than he. If you pursue an opposite course—if, because he is sensitive to the ridicule of other children, you let him remain at home, if you impress upon his mind the idea that he has a defect which must be removed, and which will be an insuperable bar to his progress in life, unless it is removed; if you allow him to perceive your constant anxiety for his care, he will get the idea that there is something peculiar in his case—that he is marked out from mankind, as if the seal of Cain were set upon his brow; and that until he is freed from his curse, he can never associate with his fellows without shame.

On the contrary, you should direct your principal attention to convince him that his fate is not an uncommon one; that defects and diseases are the common lot, and assigned for wise purposes. In as far as you direct his attention to his defect at all, let it be with this object—to convince him that it is not an evil. Teach him resignation to the will of God. Impress upon his mind above all things, that he is under the constant protection of a being who knew what was best for him, and who has placed him in the condition and under the circumstances best adapted for his welfare. Priestly attributes the greatest blessings he enjoyed to his impediment of speech; and others may in like manner trace to the same cause, their preservation from much evil, and their possession of much happiness. Our greatest felicity is often produced by what we regard at the time as our greatest misfortunes.

When he begins to feel the importance of a free use of speech (and he may feel the importance of it without being morbidly sensitive on the subject,) and disposed to enter upon a laborious course of discipline, seek out a person who has experience in the treatment of impediments of speech. Place him under his care, and if he is benefited, do not remove him and think to perfect the cure yourself. Three months is a very short time for him to remain under the superintendence of an instructor; six months is better, and where it is practicable he should remain a year. If this interferes with his other studies, it is of no consequence. He will derive benefit enough to compensate for the loss. The age I would fix upon for this trial, should be from eight to twelve. Some children, however, are as mature at the former age as others at the latter. At this period, the loss of a year's study may perchance be a gain. To a child of nervous habits, the time allowed from his instruction in speaking, may be much better employed in acquiring health and vigour, by play and exercise, than in study. But if the child is not disposed to enter into this course, if he is irritable and indocile, and regards it merely as an irksome task, it will be better to wait till a more advanced age shall convince him of its importance. Otherwise we run the risk of increasing his irritability and sensitiveness.

Moral management is, therefore, all important. In most cases it will alone be sufficient to effect a cure; and in cases where it does not, it will render the cure easy to a competent instructor. I would again urge the impropriety of subjecting the patient to a new trial if the first fails. I would urge most strenuously the necessity of leading him to the choice of that pursuit in which his defect shall afford the smallest obstacle to his progress. He is to be taught to look upon it as a necessary evil, and to shape his course accordingly. He is not to be led to bear it in his mind as the prime obstacle to his success, which must be removed before he can be happy. The molehill is thus magnified into a mountain. Whatever side he looks upon, his impediment rises up before him, shutting him out from the road to distinction. It comes to occupy so large a share of his attention that he becomes a monomaniac: on this subject, he is actually insane: there is this little diseased spot in his mind: fortunate will it be for him if it does not affect the whole; if the gangrene do not extend over all his feelings.

To the person whose age renders him the director of his own course, I would give the same directions. The same rules that must guide parents in the management of children, should guide him in the work of self-education. The first work the stammerer has to accomplish is the regulation of his mind; the acquisition of perfect self-command and of mental calmness. When this is done, the rest is easy. Until it is done, it is in vain for him to attempt by physical means to overcome his defect of utterance. The first embarrassment he meets with may cause its return. When he has brought himself to feel his impediment less keenly; to be less morbidly susceptible on the subject; then, if he is not already cured, let him apply to a person experienced in the treatment of stammering. If he meets, there, others who are afflicted as he is, it is all the better; he will no longer look upon his case as a peculiar one; and if he sees others whose impediments are worse than his, it will give him additional courage.

But great labour and perseverance are necessary in the employment of the physical means, in overcoming the perverse habits of the organs, and training them to articulate correctly. I would advise him, if it be possible, to pursue the method in the place of his usual residence, and while he continues his ordinary employments. An individual may leave his customary abode and pursuits, and go to a neighbouring town or city for his cure. His ordinary trains of association will be broken off, and the new mode of speech will be more readily adopted while he remains absent. But the moment he returns; the moment he resumes his former avocations, and is subjected to his customary objects of anxiety, his former mode of speech returns.

Whatever method may be employed for the relief of this affection, no permanent advantage will be gained, in the majority of cases, unless resolutely persevered in for one or two years. With this perseverance, it may be cured with as much certainty as any other chronic disorder, and this not by any new or patent method, but simply by attention to the course I have described.

*American Journal of the Medical Sciences.* No. 41.

